# Migration and health research: past, present, and future

**DOI:** 10.1186/s12889-023-16363-7

**Published:** 2023-07-25

**Authors:** Shira M Goldenberg, Florian Fischer

**Affiliations:** 1 Division of Epidemiology and Biostatistics, School of Public Health, San Diego State University, 9500 Campanile Drive, 92182-4162 San Diego, CA USA; 2 Institute of Public Health Charité – Universitätsmedizin Berlin, Charitéplatz 1, 10117 Berlin, Germany

## Abstract

Global migration continues to rise at unprecedented rates. Migrants are an extremely heterogeneous group and face diverse health needs related to infectious diseases, sexual and reproductive health, non-communicable diseases, and healthcare access across the whole lifespan. In this editorial, we set the context and invite contributions for a collection on ‘Migration and health’ at *BMC Public Health.*

Human migration is essential to growing economies and represents a critical part of the social and cultural fabric of our societies [1]. Global migration continues to rise at unprecedented rates, fueled by ’push’ factors such as extreme weather events caused by climate change, conflict and violence, political instability, and poverty, as well as ‘pull’ factors including kinship networks and a desire for social and economic mobility. In 2019, the number of international migrants worldwide – people residing in a country other than their country of birth – reached its highest level, at 272 million [2].

Migrant populations are extremely diverse. The International Organization on Migration defines migrants as those who move away from their place of usual residence, whether within a country or across an international border, temporarily or permanently, and for a variety of reasons [3]. The term includes those who migrate voluntarily as well as forced migrants, and encompass refugees, asylum-seekers, those with and without formal immigration status/documentation, and those who migrate for family or economic reasons. Importantly, there is no legally or universally accepted definition for ‘migrants’, as opposed to ‘refugees,’ who are defined in legal terms in the 1951 Refugee Convention [4]. In large part owing to the nature of the drivers of migration – which include fleeing from persecution, oppression, and violence – marginalized and vulnerable populations are overrepresented among migrants, including sexual and gender minorities, women, pregnant people, and unaccompanied children and adolescents.

Migrants face a broad array of healthcare needs and inequities, including those related to infectious diseases, sexual and reproductive health, chronic diseases, mental health, and violence. While there are many social and economic benefits of migration both among those who migrate and society, migrants face disproportionate exposure to social and structural factors that increase risk of adverse health outcomes in countries of origin, during transit, and in destination communities. These include conflict, trauma, violence, and other human rights violations; exclusionary migration policies; unsafe/inadequate shelter, food, and housing; racism, discrimination, and stigma; labour market exclusion; and exclusion and barriers to healthcare and other services [1, 5, 6]. In destination settings, health inequities may be compounded by language barriers, difficulties navigating health and social services, lack of social support, racism and discrimination, and socio-economic marginalization [1, 5, 7].

The COVID-19 pandemic shone a spotlight on these health inequities and access barriers faced by migrants, and has unfortunately exacerbated many of these concerns [7]. Migrants are overrepresented in precarious employment sectors, as well as those that were hardest-hit by COVID-19 in many places (e.g., agriculture, factories), and concurrently have consistently lower access to health services in most countries; [8] this has translated to increased prevalence and transmission of COVID-19 among migrants in many settings globally [9]. COVID-19 and other health inequities faced by migrants during the pandemic have been further compromised by administrative, financial, legal, and language barriers to accessing the health system, as well as shortages in medicines and healthcare facilities – quite apart from access to disease prevention and health promotion [8]. Despite all of these challenges and their structural origins, migrants are often vilified and stigmatized on the basis of misguided and xenophobic beliefs and biases that conflate migration with disease transmission – much of which is fostered by political strategies and rhetoric. A growing body of research recognizes the ways in which social and structural factors (e.g., violence, language barriers, criminalization of migrants, health insurance access, stigma, racism, and discrimination) underpin differences in the health of migrants vs. local-born populations. More research of this nature is needed to inform sustainable and pragmatic interventions that address these “upstream” determinants, particularly as it relates to the intersections between immigration policies, racism and discrimination, and health impacts.

In recent years, migration policies in a number of settings have become increasingly xenophobic and restrictive – for example, in the United States, both Republican and Democratic administrations have implemented draconian and restrictive laws aiming to prevent entry and processing of asylum-seekers, all of which are deeply antithetical to public health and human rights principles and commitments. Such policies are in stark contrast to commitments and aspirations laid out in key global policy instruments, including the Global Compact on Migration [10] and target 10.7 of the 2030 Agenda for Sustainable Development in which Member States committed to cooperate internationally to facilitate safe, orderly and regular migration. Within this policy climate, it is essential to produce research that evaluates the impact of such policies and practices on migrant health outcomes, access to care, and related human rights concerns.

Against this backdrop, the aim of the ‘Migration and health’ collection at *BMC Public Health* is to bring together research on the health inequities and care access faced by diverse migrant populations around the globe, as well as to feature research highlighting strategies for improving the health of migrants through promising evidence-based interventions. We encourage articles that advance our understanding of the myriad health needs faced by diverse migrant populations globally, including those related to infectious diseases, sexual and reproductive health, non-communicable diseases, mental health, and health care access. Contributions that consider changes in the health status, needs, and determinants of health among migrants across the stages of migration – from countries of origin to the transit stage and receiving communities – are especially encouraged, as are those that examine or identify evidence-based strategies to intervene upon the social and structural drivers of migrant health inequities.

## Data Availability

N/A.

## References

[1] Orcutt M, Spiegel P, Kumar B, Abubakar I, Clark J, Horton R (2020). Lancet Migration: global collaboration to advance migration health. The Lancet.

[2] United Nations. Migration. United Nations. 2023. https://www.un.org/en/global-issues/migration (accessed June 2, 2023).

[3] Key Migration Terms. International Organization for Migration. https://www.iom.int/key-migration-terms (accessed June 2, 2023).

[4] UN General Assembly (1951). Convention relating to the Status of Refugees.

[5] Machado S, Goldenberg S (2021). Sharpening our public health lens: advancing im/migrant health equity during COVID-19 and beyond. Int J Equity Health.

[6] Juárez SP, Honkaniemi H, Dunlavy AC (2019). Effects of non-health-targeted policies on migrant health: a systematic review and meta-analysis. The Lancet Global Health.

[7] Orcutt M, Patel P, Burns R (2020). Global call to action for inclusion of migrants and refugees in the COVID-19 response. The Lancet.

[8] Kluge HHP, Jakab Z, Bartovic J, D’Anna V, Severoni S (2020). Refugee and migrant health in the COVID-19 response. Lancet.

[9] Wiedmeyer M, Goldenberg S, Peterson S (2023). SARS-CoV-2 testing and COVID-19–related primary care use among people with citizenship, permanent residency, and temporary immigration status: an analysis of population-based administrative data in British Columbia. Can J Public Health.

[10] United Nations, GLOBAL COMPACT FOR SAFE, ORDERLY AND REGULAR, MIGRATION. 2018; published online July 13. https://refugeesmigrants.un.org/sites/default/files/180713_agreed_outcome_global_compact_for_migration.pdf.

